# Water Sorption Isotherm of Pea Starch Edible Films and Prediction Models

**DOI:** 10.3390/foods5010001

**Published:** 2015-12-24

**Authors:** Bahareh Saberi, Quan V. Vuong, Suwimol Chockchaisawasdee, John B. Golding, Christopher J. Scarlett, Costas E. Stathopoulos

**Affiliations:** 1 School of Environmental and Life Sciences, University of Newcastle, Ourimbah NSW 2258, Australia; bahareh.saberi@uon.edu.au (B.S.); vanquan.vuong@newcastle.edu.au (Q.V.V.); csuwimol@gmail.com (S.C.); john.golding@dpi.nsw.gov.au (J.B.G.); 2 NSW Department of Primary Industries, Ourimbah NSW 2258, Australia; 3 Division of Food and Drink, School of Science, Engineering and Technology, University of Abertay, Dundee DD1 1HG, UK

**Keywords:** pea starch, edible film, moisture sorption isotherm, modeling

## Abstract

The moisture sorption isotherm of pea starch films prepared with various glycerol contents as plasticizer was investigated at different storage relative humidities (11%–96% RH) and at 5 ± 1, 15 ± 1, 25 ± 1 and 40 ± 1 °C by using gravimetric method. The results showed that the equilibrium moisture content of all films increased substantially above *a_w_* = 0.6. Films plasticized with glycerol, under all temperatures and RH conditions (11%–96%), adsorbed more moisture resulting in higher equilibrium moisture contents. Reduction of the temperature enhanced the equilibrium moisture content and monolayer water of the films. The obtained experimental data were fitted to different models including two-parameter equations (Oswin, Henderson, Brunauer–Emmitt–Teller (BET), Flory–Huggins, and Iglesias–Chirife), three-parameter equations Guggenhiem–Anderson–deBoer (GAB), Ferro–Fontan, and Lewicki) and a four-parameter equation (Peleg). The three-parameter Lewicki model was found to be the best-fitted model for representing the experimental data within the studied temperatures and whole range of relative humidities (11%–98%). Addition of glycerol increased the net isosteric heat of moisture sorption of pea starch film. The results provide important information with estimating of stability and functional characteristics of the films in various environments.

## 1. Introduction

There has been an increased effort to reduce environmental impacts by using biodegradable polymers for packaging purposes, where edible coatings and films from renewable materials have been developed to improve the quality and shelf life of fresh and processed fruits and vegetables [1,2]. A main role of edible films is to decrease water loss between the food and the environment [3]. The macromolecular structure and permeability characteristics of most biodegradable films change with alterations in the relative humidities (RH) of the storage atmosphere [4]. The water binding capability of the films at a specific environmental relative humidity can be analyzed by water sorption isotherms [5]. Water sorption isotherm explains the equilibrium moisture content as a function of water activity (*a_w_*) at a constant temperature and pressure [6].

In most hydrophilic films, water functions as a plasticizer, and the relative humidity of environment determines water adsorption and desorption of films [7]. The moisture sorption behavior of foods is described by several mathematical models, some of them are according to principles of the sorption mechanism and others categorized into empirical and semi-empirical models [8,9]. There is no unique sorption isotherm model for different foods, since the single components of food products have particular hydroscopic characteristics and may change the structure or composition of the food effect on the moisture sorption isotherm [10]. Consequently, it is essential to seek for the most suitable isotherm equation for a particular biopolymer film. Several studies have investigated the sorption isotherms of starch biodegradable films [3,4,11,12,13,14,15,16,17,18]. However, there is still a lack of information on the effects of water and glycerol on the moisture sorption isotherm of pea starch films, which provides better understanding of the role of water in edible biopolymer films. The information of moisture sorption properties of edible films would assure to appropriately identify the circumstances of storage and packaging, to forecast shelf life, and to predict the physicochemical modifications in the processing of product.

This research aimed to study the moisture sorption isotherm of pea starch films with various glycerol concentrations (0%, 15%, 25% and 35% *w*/*w*) at different storage temperatures, and to fit the experimental data with prediction models.

## 2. Experimental Section

### 2.1. Materials

Canadian non-GMO yellow pea starch with 13.2% moisture, 0.2% protein, 0.5% fat and 0.3% ash, was used in all experiments (supplied by Yantai Shuangta Food Co., Jinling Town, China). All other chemicals were purchased from Merck Millipore Pty. Ltd., Victoria, Australia.

### 2.2. Preparation of Film

Aqueous dispersion (5%, *w*/*w*) of pea starch was prepared, and glycerol (plasticizer) was added to the dispersions at 0%, 15%, 25% and 35% (*w*/*w*, plasticizer/starch). The dispersions were heated in a water bath at 90 °C for 20 min with agitation to allow complete gelatinization of the starch. After gelatinization, the starch solutions were cooled to 50 to 60 °C. All the films were obtained by casting method; approximately 20 g of filmogenic suspensions were poured onto Petri dishes (10 cm in diameter). Films were formed by drying at 25 °C in an oven until reaching constant weight (about 24 h) [19].

### 2.3. Moisture Sorption Isotherm

Water sorption isotherms were measured through a gravimetrical method by exposing samples in the presence of different saturated salt solutions, which have recognized relative humidity at each specific temperature. Their *a_w_* at 5, 15, 25 and 40 °C were taken from Labuza *et al.* [20] and Rizvi [21] and were presented in Table 1. Each film (40 × 15 mm^2^) was pre-dried for 20 days over 0% RH at 25 °C to begin the sorption experiment. Samples of pre-dried sheets of 40 × 15 mm^2^ were put in small plastic cups and placed on mesh inside plastic jars containing the selected saturated solutions. The jars were then tightly closed and placed in a temperature-controlled chamber at 5, 15, 25 and 40 °C. The weight of each sample was checked using an analytical balance (with the precision of 0.0001 g) firstly after three days and then at one-day intervals until constant weight was achieved. Equilibrium was considered to be achieved when the difference between three consecutive sample weights was <1.0 mg per g of dry solid. The moisture content of the equilibrated samples was calculated after drying at 105 °C during 4 h. All tests were conducted in triplicate.

**Table 1:** The water activities (*a_w_*) of saturated salt solutions at 5, 15, 25 and 40 °C.*

Salt	5 °C	15 °C	25 °C	40 °C
LiCl	0.113	0.113	0.113	0.112
CH_3_COOK	0.291	0.234	0.225	0.216
K_2_CO_3_	0.431	0.432	0.432	0.423
Mg(NO_3_)_2_	0.589	0.559	0.529	0.484
NaNO_2_	0.693	0.693	0.654	0.628
NaCl	0.757	0.756	0.753	0.747
KCl	0.876	0.859	0.843	0.823
KNO_3_	0.963	0.954	0.936	0.890

* Source: Labuza, Kaanane and Chen [20] and Rizvi [21].

### 2.4. Mathematical Modeling

In this study the recognized Brunauer–Emmitt–Teller (BET) (Equation (1)), Guggenhiem–Anderson–deBoer (GAB) (Equation (2)), Peleg (Equation (3)), Oswin (Equation (4)), Ferro–Fontan (Equation (5)), Henderson (Equation (6)), Lewicki (Equation (7)), Iglesias–Chirife (Equation (8)) and Flory–Huggins (Equation (9)) equations were employed to fit the experimental data. These models are explained and rearranged as given below [3,18,22,23,24]:

(1) $$\text{M}=\frac{{\text{M}}_{0}.\text{ C}.{\text{a}}_{\text{w}}}{\left(1-{\text{a}}_{\text{w}}\right)\left(1+\text{C}.{\text{a}}_{\text{w}}-{\text{a}}_{\text{w}}\right)}$$

(2) $$\text{M}=\frac{M_{0}.C.K^{\prime }.a_{w}\;}{\left(1\;-\;K^{\prime }.\;a_{w}\right)\left(1-K^{\prime }.a_{w}\;+\;C.K^{\prime }.a_{w}\right)}$$

(3) $$\text{M}=K_{1}a_{w}^{n_{1}}+\;K_{2}a_{w}^{n_{2}}$$

(4) $$\text{M}=K_{0}{\left(\frac{a_{w}}{1-a_{w}}\right)}^{n_{0}}$$

(5) $$\text{M}={\left[\frac{\gamma }{\text{ln}\left(\frac{\alpha }{a_{w}}\right)}\right]}^{\frac{1}{r}}$$

(6) $$\text{M}={\left[-ln\frac{\left(1-a_{w}\right)}{A}\right]}^{\frac{1}{B}}$$

(7) $$\text{M}=\left[\frac{F}{{\left(1\;-\;a_{w}\right)}^{G}}\right]-\left[\frac{F}{\left(1\;+\;{a_{w}}^{H}\right)}\right]$$

(8) $$\text{M}=\text{A}\left[\frac{a_{w}}{\left(1-a_{w}\right)}\right]+B$$

(9) $$\text{M}=\text{A exp}\left(Ba_{w}\right)$$

where M is the equilibrium moisture content (% db); M_0_ is the monolayer moisture content; *a_w_* is the water activity; and C, K′, K_1_, K_2_, n_1_, n_2_, K_0_, γ, α, r, n_0_, A, B, F, G and H are model constants. Fitting of experimental data into the above equations was done using regression analysis MS Excel software (Microsoft Office, 2010) [3].

The suitability of the equations was estimated and compared using the correlation coefficient (*R*^2^) and the mean relative percentage deviation modulus (M_e_) [25]:

(10) $${\text{M}}_{\text{e}}=\frac{100}{n}\sum_{i=1}^{n}\frac{\left|M_{i,exp}-M_{i,pre}\right|}{M_{i,exp}}$$

where M_i,exp_ is the experimental value, M_i,pre_ is the predicted value, and n is the population of experimental data.

### 2.5. Determination of the Net Isosteric Heat of Sorption

The net isosteric heat of sorption is a differential molar quantity based on the temperature dependence of the isotherm, which displays the energies for water molecules binding at a particular hydration level [26]. The net isosteric heat of sorption $\left(q_{n}^{s}\right)$, represents the difference between the isosteric heat (Q_s_) and pure water vaporization energy (L_r_), was determined using the Clausius–Clapeyron equation:

(11) $${\left[\frac{dln\left(a_{w}\right)}{dT}\right]}_{w}=\frac{q_{n}^{s}}{RT^{2}}$$

where *a_w_* is the water activity at the absolute temperature *T* (in kelvins) and *R* is the universal gas constant (8.314 J/mol K). Through Equation (11), $q_{n}^{s}$ may be established by plotting ln(*a_w_*) at a specific moisture content *vs.* 1/T and measuring the slope [27].

### 2.6. Statistical Analysis

Glycerol was used in four levels of 0%, 15%, 25%, and 35% and replicated three times. All experiments were performed in a randomized design. Analysis of variance was carried out and the results were separated using the Multiple Ranges Duncan’s test (*p* < 0.05) using statistical software of Statistical Package for Social Science 16 (SPSS, Inc., Upper Saddle River, NJ, USA). All tests were performed at least in triplicate.

## 3. Results and Discussions

### 3.1. Moisture Sorption Isotherm

**Figure 1:**
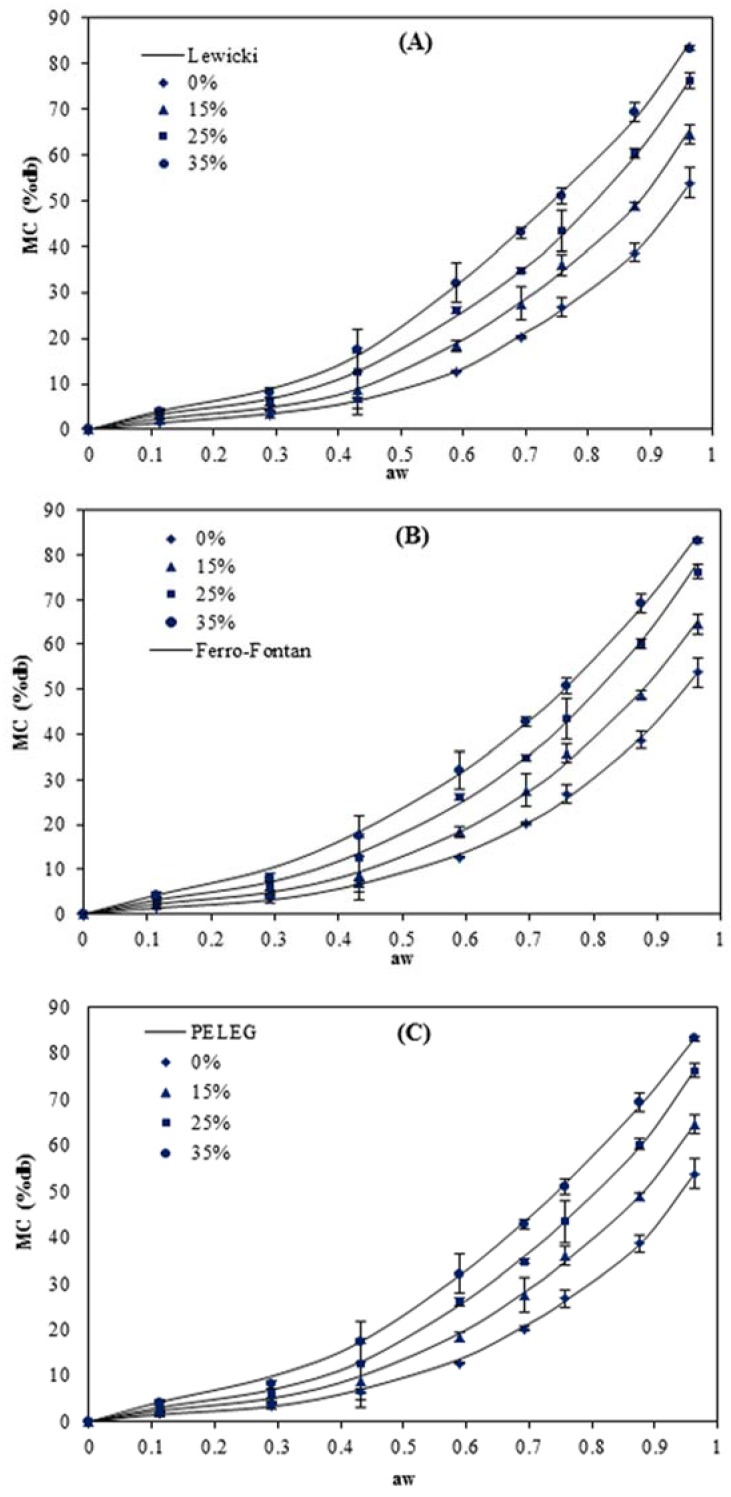
Equilibrium moisture sorption isotherm of pea starch film with different glycerol content (*w*/*w*) at 5 °C. The symbols are experimental data and the lines are from the equations obtained by fitting the experimental data to Lewicki (**A**), Ferro–Fontan (**B**) and PELEG (**C**) equations.

**Figure 2:**
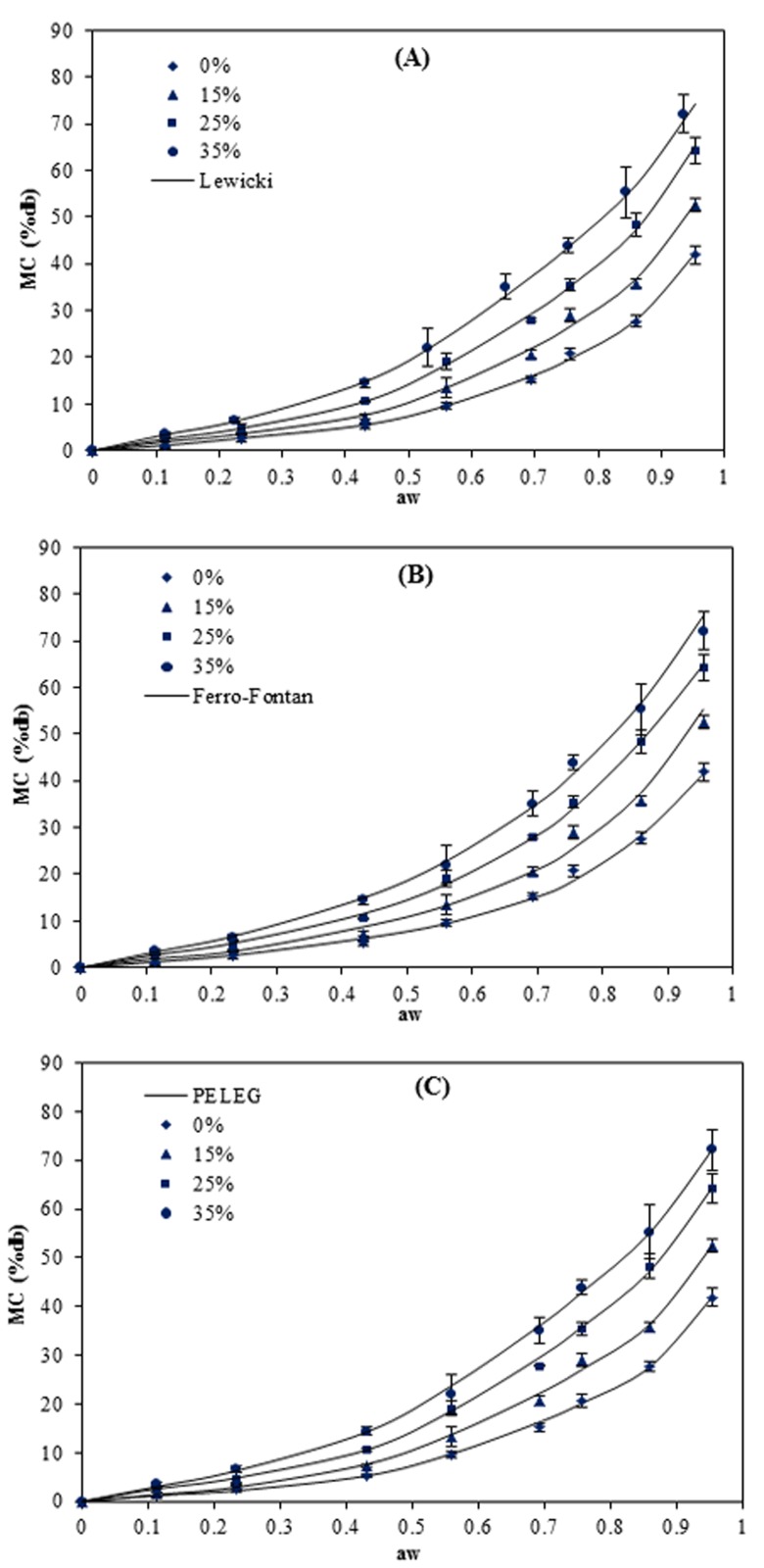
Equilibrium moisture sorption isotherm of pea starch film with different glycerol content (*w*/*w*) at 15 °C. The symbols are experimental data and the lines are from the equations obtained by fitting the experimental data to Lewicki (**A**), Ferro–Fontan (**B**) and PELEG (**C**) equations.

**Figure 3:**
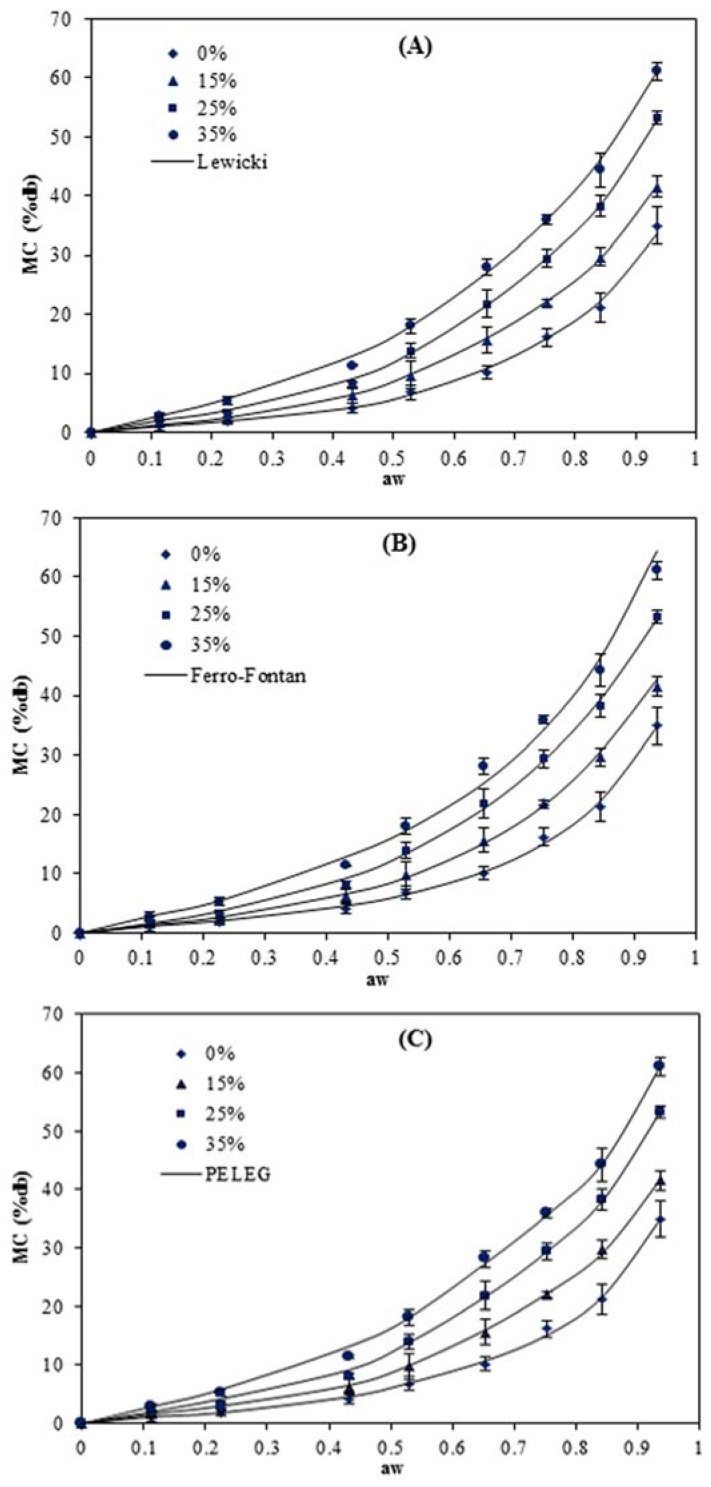
Equilibrium moisture sorption isotherm of pea starch film with different glycerol content (*w*/*w*) at 25 °C. The symbols are experimental data and the lines are from the equations obtained by fitting the experimental data to Lewicki (**A**), Ferro–Fontan (**B**) and PELEG (**C**) equations.

**Figure 4:**
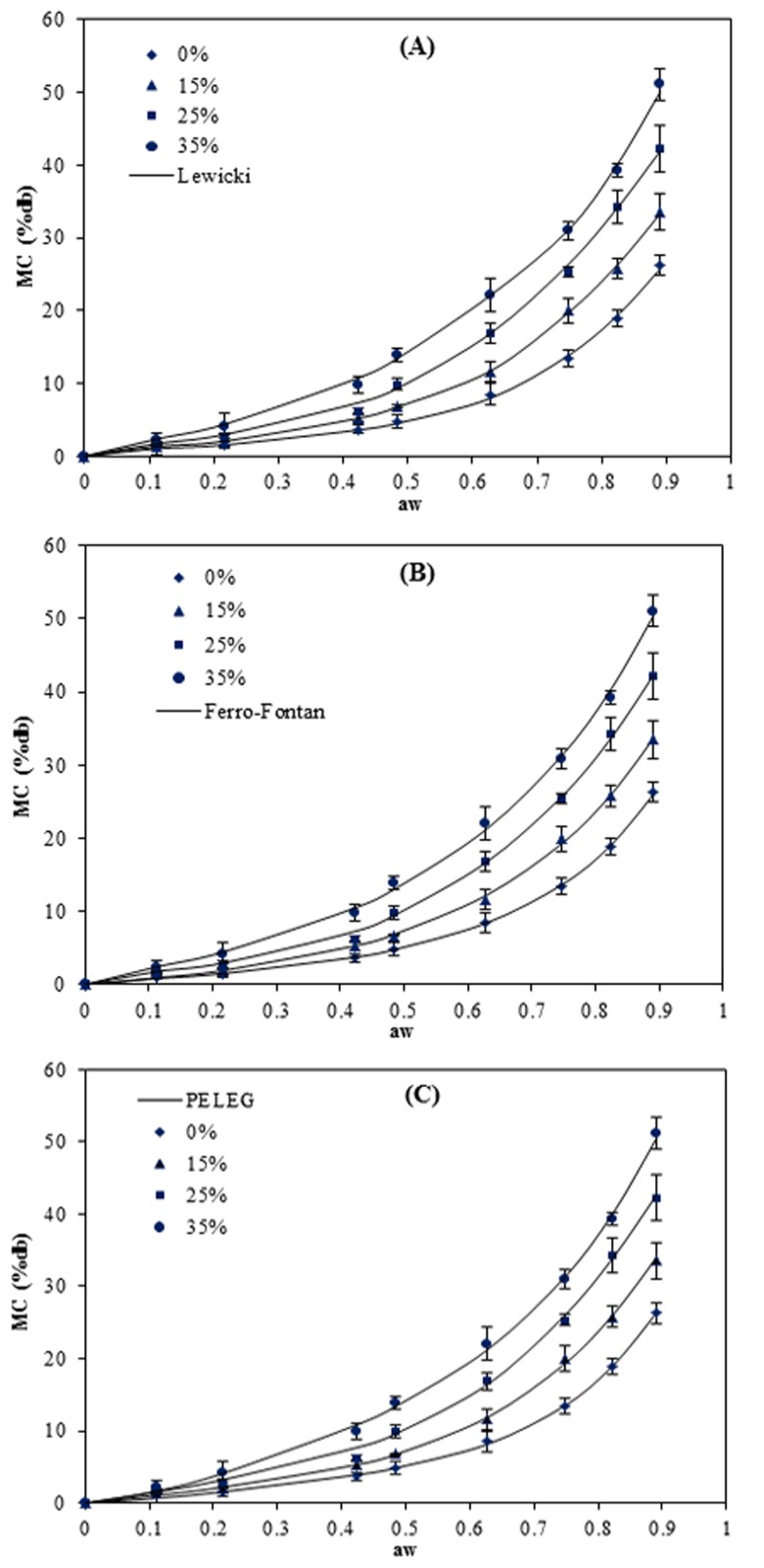
Equilibrium moisture sorption isotherm of pea starch film with different glycerol content (*w*/*w*) at 40 °C. The symbols are experimental data and the lines are from the equations obtained by fitting the experimental data to Lewicki (**A**), Ferro–Fontan (**B**) and PELEG (**C**) equation.

Moisture absorption is an important indicator of the sensitivity of material to moisture. The physical and barrier characteristics of starch-based films can be significantly affected by moisture content [15]. The moisture sorption curves of the pea starch films at 5, 15, 25 and 40 °C are presented in Figure 1, Figure 2, Figure 3 and Figure 4. The results show that these responses were a sigmoidal shape (Type III) consistent with the classification of Al-Muhtaseb *et al.* [28]. The J-shaped isotherm of glycerol-plasticized films has been reported by Enyinnaya Chinma *et al.* [29] and Coupland *et al.* [30]. The slope of the isotherms for pea starch films was smaller at lower *a_w_* (less than 0.60), with the raising in *a_w_* the slope increased quickly, which brought about large moisture adsorption with any increase in relative humidity [30]. At lower relative humidities, water strongly adsorbed to the binding sites of the film surface, while by increasing moisture content, owing to the swelling of the hydrophilic network of films [31], more new sites for water were available to bind, causing higher moisture content (MC) [32]. This is typical behavior of hydrophilic substances and is shown in water sorption isotherms of pea starch films, where similar results have been reported for other starch edible films [3,5,14,15,16,18,33,34].

Starch films are often semicrystalline, containing both amorphous and crystalline phases, similar to starch granules [35]. Hydrogen-bonding of water molecules to the accessible hydroxyl groups in the amorphous areas and on the surfaces of the crystallites, are responsible for starch sorption isotherm [36]. The amorphous regions show more inclination to water diffusion than the crystalline regions. Therefore, water influences the structure acting as a plasticizer of the amorphous regions. At low water activity, the plasticizing effect is very small and the movement of the amorphous regions is limited. While, at higher water activity, the accessibility of the hydroxyl groups to the water molecules increases due to the swelling of the biopolymer and reduction of crystallinity degree, so there is a rise in accessibility of the polar groups to the water molecules [22].

The results also showed that the MC decreased with increasing temperature, at the same *a_w_*, for each film, indicating that the starch films become less hygroscopic. At higher temperatures, the attraction forces between molecules reduce owing to an increase in kinetic energy of water molecules. So, at low temperatures, water molecules with lower energy levels are easily bound to available binding sites of the surface [22]. In addition, the incorporation of glycerol improved the capacity of films to absorb more water. In the absence of glycerol, the crystalline fraction of starch films holds a specific amount of water linked by hydrogen bonds, while amorphous regions have capacity to absorb relatively high amount of water molecules [37]. By incorporation of glycerol, the crystallization development may be partially prevented, because it can disturb the configuration of polymeric chains by interfering with amylose packing through the formation of glycerol–starch and glycerol–water interactions [38]. The increase of glycerol content resulted in weakening the cohesiveness of the polymer structure, creating the polymer network with greater interchain distances [39], thus facilitating more water molecules immobilizing into the pea starch film matrix [40]. The strong tendency of glycerol to water was associated with its high polarity [41]. Glycerol molecules, therefore, entrapped a large amount of water molecules inside the starch polymer matrix by increasing the free volume of the starch molecular network and flexibility of the polymeric chains [18]. Glycerol as a water-holding agent contributes to the formation of more hydrogen bonds in the film matrix and increases the MC in the film.

### 3.2. Modeling of Sorption Isotherms

The constants of the sorption models for pea starch films with different contents of glycerol, together with the correlation coefficient (*R*^2^) and the mean relative percentage deviation modulus (M_e_) are shown in Table 2, Table 3, Table 4 and Table 5.

**Table 2:** Estimated model constants and values of coefficients and mean relative percentage deviation moduli for different pea starch films at 5 °C.

Model Constants		Glycerol			
		0%	15%	25%	35%
**BET (0.11–0.50)**	m_0_	0.061	0.086	0.123	0.149
	C	6.228	4.276	3.159	3.090
	M_e_	17.813	17.014	12.910	11.407
	*R*^2^	0.914	0.939	0.976	0.978
**GAB (0.11–0.96)**	m_0_	0.105	0.128	0.153	0.200
	C	2.096	1.825	1.779	0.632
	K	0.893	0.888	0.848	0.670
	M_e_	9.688	9.669	7.352	2.273
	*R*^2^	0.993	0.992	0.989	0.999
**PELEG (0.11–0.96)**	k_1_	0.641	0.725	0.898	0.986
	k_2_	0.500	0.666	0.794	0.808
	n_1_	2.699	2.547	2.339	1.971
	n_2_	42.861	44.040	64.782	53.221
	M_e_	2.980	4.108	3.363	2.433
	*R*^2^	1.000	1.000	0.999	0.999
**Oswin (0.11–0.96)**	K_0_	0.165	0.194	0.259	0.331
	n_0_	0.410	0.411	0.371	0.317
	M_e_	36.746	34.435	34.668	36.267
	*R*^2^	0.970	0.975	0.965	0.934
**Ferro–Fontan (0.11–0.96)**	γ	0.070	0.108	0.098	0.125
	α	1.162	1.124	1.248	1.291
	r	0.844	0.958	0.791	0.796
	M_e_	5.464	6.662	6.739	1.908
	*R*^2^	0.994	0.992	0.989	0.998
**Henderson (0.11–0.96)**	A	11.349	12.828	20.319	37.910
	B	0.418	0.408	0.411	0.426
	M_e_	39.758	38.581	40.850	42.510
	*R*^2^	0.970	0.973	0.957	0.917
**Lewicki (0.11–0.96)**	F	0.489	0.513	0.761	1.484
	G	0.198	0.221	0.168	0.058
	H	3.146	2.675	2.467	3.105
	M_e_	1.908	1.465	2.107	0.895
	*R*^2^	1.000	1.000	0.999	1.000
**Iglesias–Chirife (0.11–0.65)**	A	0.096	0.115	0.148	0.181
	B	1.572	1.933	3.378	5.340
	M_e_	13.622	11.033	5.988	5.072
	*R*^2^	0.987	0.992	0.997	0.996
**Flory–Huggins (0.11–0.96)**	A	0.999	1.210	2.655	4.270
	B	4.462	4.428	3.752	3.097
	M_e_	14.833	15.236	8.319	4.775
	*R*^2^	0.985	0.981	0.984	0.998

**Table 3:** Estimated model constants and values of coefficients and mean relative percentage deviation moduli for different pea starch films at 15 °C.

Model Constants		Glycerol			
		0%	15%	25%	35%
**BET (0.11–0.50)**	m_0_	0.044	0.061	0.083	0.116
	C	13.807	8.780	8.314	7.480
	M_e_	8.785	13.334	6.840	5.579
	*R*^2^	0.952	0.932	0.977	0.993
**GAB (0.11–0.96)**	m_0_	0.079	0.098	0.102	0.180
	C	6.223	2.680	2.510	3.144
	K	0.918	0.922	0.912	0.869
	M_e_	10.344	7.961	7.089	5.042
	*R*^2^	0.990	0.995	0.989	0.993
**PELEG (0.11–0.96)**	k_1_	0.549	0.679	0.706	0.900
	k_2_	0.782	0.827	1.083	1.519
	n_1_	2.837	2.826	2.445	2.210
	n_2_	50.955	78.354	46.823	44.471
	M_e_	4.353	4.217	4.270	3.349
	*R*^2^	1.000	0.999	0.998	0.999
**Oswin (0.11–0.96)**	K_0_	0.127	0.157	0.187	0.268
	n_0_	0.479	0.466	0.458	0.396
	M_e_	25.312	24.900	17.415	23.917
	*R*^2^	0.984	0.981	0.989	0.977
**Ferro–Fontan (0.11–0.96)**	γ	9.082	13.138	25.611	13.993
	α	1.077	1.076	1.052	1.193
	r	1.052	1.101	1.265	0.901
	M_e_	7.906	6.875	6.225	4.475
	*R*^2^	0.991	0.994	0.993	0.993
**Henderson (0.11–0.96)**	A	7.431	9.028	10.555	18.236
	B	0.404	0.397	0.390	0.397
	M_e_	29.147	29.615	22.422	31.165
	*R*^2^	0.984	0.979	0.986	0.968
**Lewicki (0.11–0.96)**	F	0.237	0.352	0.296	0.569
	G	0.344	0.297	0.367	0.246
	H	0.450	2.162	0.981	2.180
	M_e_	1.664	2.624	2.133	1.708
	*R*^2^	0.999	1.000	1.000	1.000
**Iglesias–Chirife (0.11–0.65)**	A	0.071	0.085	0.095	1.426
	B	1.893	2.662	4.612	5.622
	M_e_	11.114	8.512	4.613	1.998
	*R*^2^	0.991	0.992	0.995	0.999
**Flory–Huggins (0.11–0.96)**	A	0.579	0.874	1.095	2.954
	B	4.910	4.649	4.569	3.684
	M_e_	20.798	18.667	21.679	11.052
	*R*^2^	0.977	0.972	0.972	0.987

**Table 4:** Estimated model constants and values of coefficients and mean relative percentage deviation moduli for different pea starch films at 25 °C.

Model Constants		Glycerol			
		0%	15%	25%	35%
**BET (0.11–0.50)**	m_0_	0.040	0.051	0.072	0.086
	C	12.081	11.562	10.276	8.791
	M_e_	9.321	11.223	6.201	7.380
	*R*^2^	0.942	0.928	0.978	0.974
**GAB (0.11–0.96)**	m_0_	0.057	0.082	0.097	0.158
	C	4.354	4.166	2.838	2.099
	K	0.937	0.927	0.922	0.881
	M_e_	6.643	8.005	4.774	5.993
	*R*^2^	0.997	0.997	0.996	0.997
**PELEG (0.11–0.96)**	k_1_	0.377	0.518	0.617	0.801
	k_2_	0.558	0.561	0.641	0.749
	n_1_	2.375	2.505	2.361	2.426
	n_2_	25.021	24.454	27.416	24.179
	M_e_	4.860	2.913	4.451	5.396
	*R*^2^	1.000	1.000	0.998	0.998
**Oswin (0.11–0.96)**	K_0_	0.099	0.132	0.166	0.218
	n_0_	0.525	0.517	0.490	0.450
	M_e_	15.811	20.650	16.138	21.559
	*R*^2^	0.992	0.988	0.989	0.976
**Ferro–Fontan (0.11–0.96)**	γ	7.551	7.566	12.186	8.768
	α	1.060	1.088	1.083	1.195
	r	1.081	0.961	1.047	0.811
	M_e_	3.543	5.231	3.581	4.556
	*R*^2^	0.998	0.998	0.996	0.997
**Henderson (0.11–0.96)**	A	5.654	6.870	8.716	12.130
	B	0.402	0.387	0.384	0.385
	M_e_	18.286	24.909	21.403	26.724
	*R*^2^	0.994	0.989	0.988	0.973
**Lewicki (0.11–0.96)**	F	0.198	0.301	0.308	0.587
	G	0.365	0.324	0.351	0.231
	H	3.256	3.703	2.590	3.357
	M_e_	3.364	2.682	1.054	2.841
	*R*^2^	1.000	1.000	1.000	1.000
**Iglesias–Chirife (0.11–0.65)**	A	0.059	0.081	0.096	0.117
	B	1.925	1.929	3.405	4.565
	M_e_	8.090	10.807	3.505	3.961
	*R*^2^	0.993	0.986	0.990	0.996
**Flory–Huggins (0.11–0.96)**	A	0.382	0.557	0.966	1.846
	B	5.170	5.043	4.611	4.085
	M_e_	26.223	23.089	21.889	14.115
	*R*^2^	0.978	0.983	0.981	0.992

**Table 5:** Estimated model constants and values of coefficients and mean relative percentage deviation moduli for different pea starch films at 40 °C.

Model Constants		Glycerol			
		0%	15%	25%	35%
**BET (0.11–0.50)**	m_0_	0.036	0.045	0.060	0.079
	C	11.613	10.907	10.774	9.746
	M_e_	8.712	9.245	5.241	3.756
	*R*^2^	0.956	0.939	0.988	0.988
**GAB (0.11–0.96)**	m_0_	0.050	0.068	0.082	0.121
	C	1.175	0.816	0.793	0.483
	K	0.916	0.899	0.866	0.862
	M_e_	6.474	5.899	6.447	5.112
	*R*^2^	0.995	0.996	0.998	0.996
**PELEG (0.11–0.96)**	k_1_	0.168	0.171	0.184	0.203
	k_2_	0.550	0.731	0.911	0.946
	n_1_	1.297	0.911	0.764	0.633
	n_2_	8.568	7.502	7.945	6.300
	M_e_	7.920	5.338	4.309	4.200
	*R*^2^	0.997	0.998	1.000	0.998
**Oswin (0.11–0.96)**	K_0_	0.092	0.121	0.139	0.189
	n_0_	0.561	0.553	0.564	0.503
	M_e_	17.298	22.958	13.425	17.766
	*R*^2^	0.978	0.973	0.986	0.971
**Ferro–Fontan (0.11–0.96)**	γ	3.411	3.579	5.613	6.280
	α	1.153	1.204	1.120	1.202
	r	0.731	0.649	0.813	0.735
	M_e_	5.078	5.677	5.719	4.493
	*R*^2^	0.998	0.998	0.999	0.996
**Henderson (0.11–0.96)**	A	5.016	6.034	6.422	9.177
	B	0.393	0.378	0.367	0.372
	M_e_	19.361	26.530	17.221	22.929
	*R*^2^	0.982	0.976	0.988	0.970
**Lewicki (0.11–0.96)**	F	0.682	0.948	0.717	0.515
	G	0.102	0.087	0.175	0.222
	H	9.002	8.227	9.130	2.671
	M_e_	3.778	5.264	1.486	1.308
	*R*^2^	0.999	0.998	1.000	1.000
**Iglesias–Chirife (0.11–0.65)**	A	0.057	0.075	0.75	0.102
	B	1.541	1.652	3.370	4.311
	M_e_	7.160	9.439	2.473	3.329
	*R*^2^	0.980	0.979	0.996	0.993
**Flory–Huggins (0.11–0.96)**	A	0.303	0.452	0.449	1.196
	B	5.381	5.232	5.414	4.504
	M_e_	27.921	24.716	28.821	20.898
	*R*^2^	0.988	0.991	0.989	0.990

The most recognized models for calculation of monolayer moisture content (m_0_) of foods materials are BET and GAB. The monolayer value is evidence of the quantity of water that can be bound to a single layer per gram of dry film [42]. Since the GAB model is associated with the water sorption in the multi-layer and the BET model in the first layer region, it is expected that the GAB model results in higher values of monolayer moisture content than those measured by the BET model [32]. The results showed that the water content associated to the monolayer of pea starch film without plasticizer analyzed in the range of 5–40 °C, varied from 0.061 to 0.036 g water/g dry solids, when the BET model was considered and from 0.105 to 0.050 g water/g dry solids for the GAB model. Comparing with pea starch film content 15% and 35% *w*/*w* glycerol, the monolayer moisture content varied from 0.086 to 0.045 g water/g dry solids and 0.149 to 0.079 g water/g dry solids, respectively. When the BET model was considered for the pea starch film content 15% and 35% *w*/*w* glycerol, the results were from 0.128 to 0.068 g water/g dry solids and 0.200 to 0.121 g water/g dry solids for the GAB model, all in the range of 5 to 40 °C, respectively. It is proposed that the higher plasticizer content, the more active sites were available to bind water molecules [43]. In addition, the m_0_ values for all pea starch films showed an affinity to reduce with an increase in temperature because at higher temperatures some water molecules can escape from their sorption sites [44]. The reduction in the monolayer moisture content with increasing temperature has also been observed by Su *et al.* [45], Perdomo, Cova, Sandoval, García, Laredo and Müller [25], Peng *et al.* [46], [22], and McMinn, Al-Muhtaseb and Magee [23].

In addition to m_0_, the sorption energy constant, C, is also related to the monolayer heat sorption in the BET and GAB models [47]. The difference in heat of sorption between the monolayer (*E*_1_) and the multilayer or bulk water (*E*_L_) is related to *C*_BET_ [48]. A reduction in the *C*_BET_ means a decrease in the value of *E*_1_ (for monolayer), while *E*_L_ (bulk liquid) would remain constant. In other words, the high value of this energy constant demonstrates that water molecules more strongly adsorbed in the active sites of the matrix [49]. Comparison of the pea starch films with different glycerol concentrations showed a decrease in this parameter when the glycerol content increased, suggesting that this polyol might occupy some of the sorption sites of the polymer. The difference in *C* from GAB and BET is mostly due to the fitting process; in terms of GAB it contains another parameter *K*_GAB_, contributing equally to *C*_GAB_ in the model fitting. Theoretically, the GAB equation considers the sorption energies in both monolayer and multilayer domains in comparison with BET, which examines only the sorption heat for the monolayer, supposing similar energies linked to the multilayer. So, it is expected to achieve smaller values for *C*_GAB_ compared to *C*_BET_ [49]. Another energy constant of the GAB model is K. An increase in *K*_GAB_ toward a value of 1 would suggest a smaller difference between the energy associated with the heat of sorption of the multilayer and the heat of condensation of pure water [49]. It is possible that hydroxyl groups of glycerol, on the sorption sites of the matrix, could increase the interaction energies between the water molecules, on the second and higher water layers, and the polymer. In this experiment, this value in all films formulations was near to 1 and independent of composition.

The M_e_ of the GAB and the BET model changed between 2.3%–10.3% and 3.8%–17.8%, respectively. The BET was not considered as an appropriate model for fitting the data due to its restriction to *a_w_* below 0.5. GAB model has been recognized for predicting the sorption behavior of different starchy products [4,25,50,51,52,53]. However, in this case, the modeling showed that the GAB model was not the best model for describing the sorption isotherm experimental data.

The Peleg model can be applied as an alternative to the classic BET and GAB models to correctly represent sorption isotherms, due to the presence of an extra parameter in the equation. The lack of a theoretical background in its development is the drawback of using this model to represent a fundamental prediction of the differences in sorption isotherm behavior. In this model, k_1_ is a constant associated with mass transfer, the lower k_1_, the higher the initial water adsorption rate; k_2_ is a constant connected to maximum water adsorption capability and the lower the k_2_, the higher the adsorption capacity [54]. Unplasticized pea starch film had lower k_1_ values, indicating that this film adsorbed water at a higher initial rate with increasing % RH. Pea starch films with higher values of glycerol adsorbed water slower at low values of % RH (higher k_1_ value) and showed higher k_2_ values (indicating higher water adsorption capacity), which was probably due to the plasticizing effect [13,24,54]. As it can be observed, the Peleg model provided a good fit to the experimental data in the temperature range studied with as evidenced by high *R*^2^ and low M_e_ values.

The Oswin and Henderson models were inadequate to describe the experimental data providing an average M_e_ value above 25% and 29%, respectively. The similar results have been observed by Al-Muhtaseb, McMinn and Magee [22] and Perdomo, Cova, Sandoval, García, Laredo and Müller [25]. The Flory–Huggins model also failed to represent the experimental data with M_e_ value ranging from 4.8% to 28.8%, and an average value of 16.8%. In the range of water activity 0.11< *a_w_* < 0.65, the Iglesias–Chirife model was displayed a satisfactory fit to the experimental data, providing an average M_e_ value of 7.8% for adsorption data. The results were in agreement with sorption isotherm of polyethylene oxide-corn starch blended films [3].

Among the two other models (Ferro–Fontan and Lewicki) which were applied throughout the whole range of water activity (0.11–0.96), the smallest M_e_ value (0.9%–5.3%) and higher values of *R*^2^ (0.999–1.000) at all the temperatures were achieved for the Lewicki model. Figure 1, Figure 2, Figure 3 and Figure 4 also confirmed that the Lewicki equation provided the best fit to the experimental data at all the investigated temperatures, followed by the Ferro–Fontan model. The mean relative percentage deviation modulus (M_e_), with a modulus value below 10% was indication of a close fit for experimental values [25]. The Lewicki model provided the lowest M_e_ values with average value of 3.1%, in comparison with 4.9% for the Ferro–Fontan model. McMinn, Al-Muhtaseb and Magee [23] also reported that the three parameter Lewicki model is the most adequate model for predicting the sorption properties of raw potato, potato starch, starch–sugar and starch–salt gels within the temperature studied and water activity range.

### 3.3. Net Isosteric Heat of Sorption

**Figure 5:**
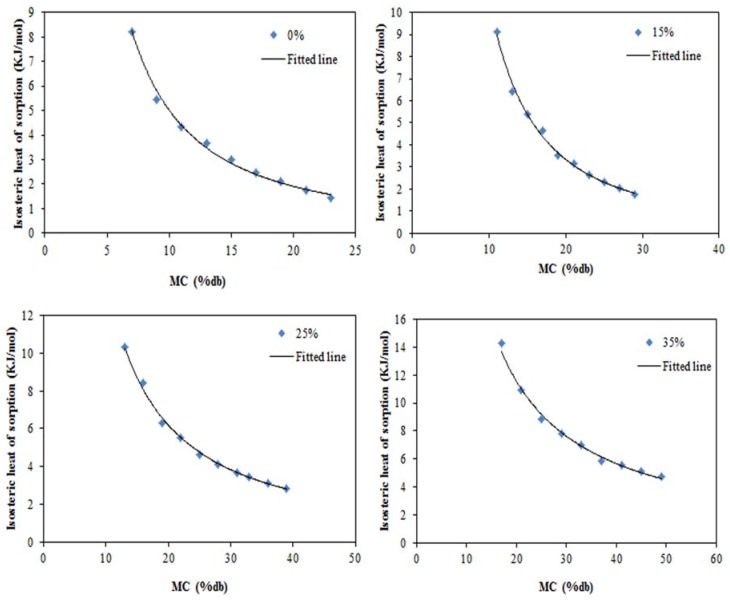
Net isosteric heat of sorption of pea starch films as a function of MC: 0% is pea starch film, 15% is pea starch film content 15% *w*/*w* glycerol, 25% is pea starch film content 25% *w*/*w* glycerol, and 35% is pea starch film content 35% *w*/*w* glycerol.

The net isoteric heat of sorption examined by applying the Clasusius–Clapeyron equation for pea starch films is exhibited in Figure 5. The net isoteric heat of sorption $\left(q_{n}^{s}\right)$, values were calculated from the slope of the plot between the values of ln(*a_w_*) and 1/T at a specific moisture content. It can be seen that the heat of sorption showed similar trend for all pea starch films. The isosteric heat is dependent on moisture content and on the energy required for sorption [55]. The amount of required energy to remove water from the solid is considered the isosteric heat of sorption, so the more tightly the water is bound, the higher is the isosteric heat of sorption [56]. The high value of the isosteric heat of sorption is a sign of the intermolecular attractive forces between the sorption sites and the water vapor at low moisture contents; this heat reduces with increase in the moisture contents [27]. At low moisture content, sorption happens on the most active sites, hydrophilic groups, while water molecules bind with less active site in higher moisture content resulting in lower isosteric heats of sorption [57,58]. The decreasing of net isosteric sorption heat with moisture content could be also associated with the interaction energy of water molecules with surface, which is decreased when coverage level of the surface is increased [59]. The net isosteric heat of sorption ranged from 8.23 kJ/mol at moisture content of 0.07 g/g dry matter to 1.43 kJ/mol at a moisture content of 0.27 g/g dry matter for control film and the corresponding value for pea starch film with 35% glycerol was 14.26 kJ/mol at moisture content of 0.17 g/g dry matter and 4.22 kJ/mol at moisture content of 0.49 g/g dry matter. The reducing behavior of the isosteric heat of sorption with increasing moisture content has been also reported in other starchy products [22,26,60,61]. The incorporation of glycerol increased the binding energy between water and the film surface led to increasing the isosteric heat of sorption. The isosteric heats *versus* moisture content results are sufficiently represented as power function of moisture content as follows:

(12) $$\text{Pea starch film}:\;q_{n}^{s}=124.77M_{e}^{-1.398},\;R_{2}=0.9912$$

(13) $$\text{Pea starch film}+15\%\text{ glycerol}:\;q_{n}^{s}=472.69M_{e}^{-1.653},\;R_{2}=0.9957$$

(14) $$\text{Pea starch film}+25\%\text{ glycerol}:\;q_{n}^{s}=213.07M_{e}^{-1.181},\;R_{2}=0.9972$$

(15) $$\text{Pea starch film}+35\%\text{ glycerol}:\;q_{n}^{s}=252.35M_{e}^{-1.029},\;R_{2}=0.9942$$

These mathematical relationships may be used to calculate the heat of sorption of pea starch films for various moisture contents.

## 4. Conclusions

The moisture sorption of the pea starch films increased with increasing water activity at different temperatures (5, 15, 25, and 40 °C) and represented a Type III isotherm. The equilibrium moisture content and monolayer moisture contents (m_0_) reduced with increases in storage temperature at constant water activity. The results showed that the glycerol concentration had a significant effect on equilibrium moisture content and monolayer moisture content (m_0_) of films. Sorption isotherm studies, with data fitted to theoretical, kinetic, semi-empirical and empirical models, showed that high correlation coefficient of determination (*R*^2^) and the lowest average relative percentage deviation modulus (M_e_) were obtained from the Lewicki model indicating that it fits the best to the experimental data followed by Ferro–Fontan and Peleg equations for all films, at all temperatures, in the whole range of water activity. The net isosteric heat of sorption of the films was measured. It was found that this heat changed inversely with variation in the amount of the absorbed moisture. These fundamental data are important in assessing applicability of starch-based edible films in food and pharmaceutical industries, due to the influence of moisture content on water vapor permeability, physical and mechanical properties of starch-based edible films.
